# Physical activity trajectories, mortality, hospitalization, and disability in the Toledo Study of Healthy Aging

**DOI:** 10.1002/jcsm.12566

**Published:** 2020-03-12

**Authors:** Juan Luis Sanchez‐Sanchez, Mikel Izquierdo, Jose Antonio Carnicero‐Carreño, Fransico José García‐García, Leocadio Rodríguez‐Mañas

**Affiliations:** ^1^ Navarrabiomed Complejo Hospitalario de Navarra‐Universidad Pública de Navarra, IDISNA Pamplona Navarra Spain; ^2^ CIBER of Frailty and Healthy Aging (CIBERFES) Instituto de Salud Carlos III Madrid Spain; ^3^ Biomedical Research Foundation Getafe University Hospital Getafe Spain; ^4^ Geriatrics Department Virgen Del Valle Hospital Toledo Spain; ^5^ Geriatrics Department Getafe University Hospital Ctra. de Toledo Getafe Spain

**Keywords:** Physical activity, Healthy aging, Trajectories, Adverse outcomes, Older adults, Mortality

## Abstract

**Background:**

Physical activity (PA) is a recognized contributor to healthy aging. However, the majority of studies exploring its associations with adverse outcomes in cohorts of older adults use single‐time PA estimates, which do not consider its dynamic nature. The aim of the present study is to explore the presence of different PA trajectories in the Toledo Study of Healthy Aging and their association with adverse outcomes. Our hypothesis is that prospectively maintaining or increasing PA is associated with a reduced risk of adverse outcomes.

**Methods:**

We used data from 1679 participants enrolled in the Toledo Study of Healthy Aging. Trajectories based on the Physical Activity Scale for the Elderly were identified using group‐based trajectory modelling. Cox and logistic regression were used to investigate associations between PA trajectories and mortality and hospitalization, and incident and worsening disability, respectively. Mortality was ascertained by linkage to the Spanish National Death Index; disability was evaluated through the Katz Index; and hospitalization was defined as the first admission to Toledo Hospital. Models were adjusted by age, sex, smoking, Charlson Index, education, cognitive impairment, polypharmacy, and Katz Index at Wave 2.

**Results:**

We found four PA‐decreasing and one PA‐increasing trajectories: high PA‐consistent (*n* = 566), moderate PA‐mildly decreasing (*n* = 392), low PA‐increasing (*n* = 237), moderate PA‐consistent (*n* = 191), and low PA‐decreasing (*n* = 293). Belonging to the high PA‐consistent trajectory group was associated with reduced risks of mortality as compared with the low PA‐decreasing group [hazard ratio (HR) 1.68; 95% confidence interval (CI) = 1.21–2.31] and hospitalization compared with the low PA‐increasing and low PA‐decreasing trajectory groups (HR 1.24; 95% CI = 1.004–1.54 and HR 1.25; 95% CI = 1.01–1.55, respectively) and with lower rates of incident [odds ratio (OR) 3.14; 95% CI = 1.59–6.19] and worsening disability (OR 2.16; 95% CI = 1.35–3.45) in relation to the low PA‐decreasing trajectory group and at follow‐up. Increasing PA during late life (low PA‐increasing group) was associated with lower incident disability rates (OR 0.38; 95% CI = 0.19–0.82) compared with decreasing PA (low PA‐decreasing group), despite similar baseline PA.

**Conclusions:**

Our results suggest that sustaining higher PA levels during aging might lead to healthy aging, characterized by a reduction in adverse outcomes. Our study supports the need for enhancing PA participation among older populations, with the goal of reducing personal and economic burden in a worldwide aging population.

## Introduction

Lifelong physical activity (PA) promotes a wide range of health benefits and has long been recognized as an important protective factor for chronic diseases.^1^, ^2^, ^3^, ^4^, ^5^, ^6^ These beneficial effects consistently translate into lower mortality rates in both men and women.^7^, ^8^, ^9^, ^10^, ^11^, ^12^

The salutary effects of PA might extend to late life, as it is known to delay the onset of disability^13^, ^14^ and to increase lifespan.^7^, ^8^, ^15^, ^16^ Furthermore, PA might be negatively associated with other adverse outcomes such as hospitalization, thereby reducing health care expenditure.^17^, ^18^ Remarkably, at advanced ages, PA levels might surpass other cardiovascular or sociodemographic risk factors that are classically associated with adverse outcomes in younger cohorts.^19^, ^20^

The World Health Organization defines ‘healthy aging’ as the process of developing and maintaining the functional ability that enables well‐being in older age.^21^ Accordingly, PA is suggested to be an important contributor to healthy aging through the maintenance and enhancement of intrinsic capacity—mental and especially physical capacities.^22^ A common methodological limitation in exploring the association between PA and adverse outcomes in older populations is the use of a single time‐point assessment of PA (primarily the baseline PA levels) as the exposure variable,^23^, ^24^, ^25^, ^26^, ^27^, ^28^, ^29^ which does not account for the dynamic nature of PA behaviours.^30^ It is plausible that prospective trajectories (patterns) of PA levels along time in late life may influence adverse outcomes distinctly as compared with cross‐sectional estimates,^31^, ^32^ a hypothesis that remains untested to our knowledge.

Some studies have recently addressed this shortcoming and used prospective PA level evolution as the exposure variable. Most of these studies employed categorical analyses with clinical or empirical cut‐points for identifying groups with different PA progression patterns.^9^, ^33^, ^34^, ^35^

Data‐driven approaches such as group‐based trajectory modelling (GBTM) have emerged as an informative and interesting analytical method that allows grouping of subjects presenting with similar baseline values and longitudinal patterns of change, in terms of their direction and magnitude, along follow‐up for a given variable within a population.^36^, ^37^ Using this methodology, some studies have shown the existence of different PA level trajectories in older adult cohorts,^30^, ^38^ and one study explored their associations with mortality in a sample of older men.^39^

The main aim of this study is to investigate the existence of different PA trajectories within the Toledo Study of Healthy Aging (TSHA) sample, a Spanish longitudinal population‐based study, and to evaluate their associations with adverse outcomes (mortality, disability onset and worsening, and hospitalization). Our hypothesis is that chronically active subjects and those maintaining PA over time will have a lower likelihood of experiencing adverse outcomes compared with consistently inactive subjects or those reducing PA levels during follow‐up and that increasing PA even at older ages promotes healthy aging, as characterized by reduced mortality, disability onset/worsening, and hospitalization rates.

## Methods

### Study design and participants

Data were taken from the TSHA study, the details of which have been reported elsewhere.^40^ Briefly, this is a population‐based prospective cohort study examining the determinants and consequences of frailty in institutionalized and community‐dwelling individuals older than 65 years living in the province of Toledo (Spain). For the present analysis, we used data from those subjects with non‐missing PA scores from the first (2006–2009) and second (2011–2013) TSHA waves and available mortality and hospitalization information at the censoring time and functional ability from the third wave (2015–2017).

The Clinical Research Ethics Committee of Toledo Hospital, Spain, approved the study protocol, and participants signed an informed consent prior to their inclusion in the study.

### Measures

#### Physical Activity Scale for the Elderly

Physical activity levels were estimated using the Physical Activity Scale for the Elderly (PASE). This questionnaire was designed to assess PA in epidemiologic studies of older people over a 1 week period. It ascertains the duration, intensity, and frequency of several activities and consists of 10 items that focus on three domains: leisure (five components), household (four), and work (one) activities. Participation in leisurely activities is recorded by frequency (e.g. never, seldom, sometimes, and often) and duration (e.g. less than an hour, 2–4 h, or >4 h); paid or unpaid work is recorded by total hours of work per week; and household activities and care for others are recorded with yes or no answers. Total PASE score is calculated by multiplying activity participation (yes/no) or the amount of time spent on each activity (hours/week) by empirically derived item weights, which are summed.^41^ In the present study, PASE was administered by interview, because this modality has proven superior reliability than self‐administration.^42^ We used PASE scores from Wave 1 and Wave 2 to construct the trajectories.

#### Mortality

Vital status was ascertained through the information provided by the Spanish National Death Index (Ministry of Health and Social Services). Participants were followed up to death or June 2018, whichever came first. The average follow‐up for mortality was 5.92 years (range 0.01–7.5 years).

#### Hospitalization

Hospitalization was ascertained by review of the Toledo Hospital Complex records and was defined as the occurrence of a first admission to the hospital during follow‐up, up to December 2016. Median follow‐up for hospitalization was 4.08 years (range 0.01–5.24 years).

#### Disability and worsening disability

The Katz Index was used to assess the functional ability in basic activities of daily living (BADLs).^43^ Incident disability was defined as the transition from a score of 6 to 5 or less in the Katz Index at follow‐up. Worsening disability at follow‐up was defined as the advent of a new difficulty in the Katz Index at follow‐up in those participants with a prevalent disability at baseline. Median follow‐up for disability onset/worsening was 2.99 years (range 2–5.4 years).

#### Covariates

We selected covariates based on the literature and the biological plausibility for confounding the main associations of interest. Age, sex, education (non‐educated, non‐finished primary education, and finished primary education/superior), and smoking status (yes/past/never) were registered during study visits. Presence of co‐morbidities was ascertained by self‐report and by checking the medical history to compute the Charlson Index score.^44^ Body mass index was computed using the standard formula (body mass × height^−2^). Cognitive status was assessed by using the Mini‐Mental State Examination (MMSE).^45^ The number of prescription and non‐prescription drugs within the Anatomical Therapeutic Chemical Classification System taken by the participant was calculated. Polypharmacy was defined as the intake of ≥5 drugs per day.^46^ All covariates were measured at Wave 2 assessment.

### Statistical analysis

#### Descriptive analysis

All analyses were performed using the R statistical environment for Windows. Mean (standard deviation) and frequency (percentage) are provided for continuous and categorical variables, respectively. Descriptive variables were compared between included and excluded subjects and between trajectories with an independent Student's *t*‐test or analysis of variance for continuous variables and the *χ* ^2^ test for categorical variables.

#### Trajectory modelling

We used a GBTM approach to identify PA trajectories within our population. This type of finite mixture model provides an empirical means of identifying clusters of individuals defined by their developmental courses for a variable over time (trajectories) within a population. Briefly, this method assumes that the general population is composed of literally distinct subpopulations that are not identifiable based on measured characteristics *ex ante*. In GBTM, each group is conceptually thought of a collection of individuals who follow approximately the same developmental trajectory, where population variability is captured by differences across groups in the shape and direction of their trajectories.^37^ First, the best model among those with different number of classes (trajectories) was identified by using Bayesian information criterion (BIC). BIC is an index used in Bayesian statistics to choose between two or more alternative models, given the data. In our study, two times the change in the BIC between models greater than 10 was used as indicative of better fit in order to compare more complex—with a greater number of trajectories—vs. more parsimonious—with a lower number of trajectories—models.^47^ Each subject was assigned to a trajectory depending upon his individual values (baseline PASE score and progression patterns). Second, average posterior probabilities of membership were computed for each group to estimate the reliability of the classification. Individual posterior probability of membership for a subject represents his probability of belonging to the group he is assigned to by previous grouping based on his individual features. Trajectory average posterior probability of membership represents its internal consistency, with higher values indicating better classification quality.^37^ We finally checked the number of subjects within each trajectory to ensure adequate sample size for assessing the subsequent risk of adverse outcomes.

#### Associations between physical activity trajectories and adverse events

Cox proportional hazards regression was used to investigate the associations between the GBTM‐derived PA trajectories and the adverse outcomes for which we had the date of occurrence (mortality and hospitalization). Logistic regression analysis was used for disability onset and disability worsening (that were registered during study visits). Multivariate trajectory models were adjusted for age and sex (Model 1), plus Charlson Index (Model 2), and additionally for baseline Katz Index, level of education, polypharmacy, cognitive status (MMSE), and smoking status (Model 3).

## Results

Baseline characteristics of the subjects within each trajectory are presented in *Table* 1; 1679 subjects (67.48% of TSHA whole sample; mean age = 74.26 ± 5.32; 41% men) had data available for the purpose of this analysis. Not‐included subjects were significantly older and had lower scores for the MMSE, Charlson Index, and Katz Index (Supporting Information, *Table S1*).

**Table 1:** Baseline characteristics of the sample

	HPAC *n* = 566; 33.7%	MPAMD *n* = 392; 23.3%	LPAI *n* = 191; 11.7%	MPAC *n* = 293; 17.5%	LPAD *n* = 237; 14.1%	Whole sample *n* = 1679	Between‐groups differences (*P* value)
Age, mean (SD)	72.37 (4.41)	73.76 (4.83)	74.94 (5.06)	72.42 (4.41)	78.9 (5.56)	74.94 (5.06)	<0.001
Men, *n* (%)	287 (50.71)	156 (39.79)	107 (56.02)	57 (19.45)	94 (39.66)	701 (41.74)	<0.001
BMI, mean (SD), kg m^−2^	28.9 (4.1)	29.3 (4.5)	30.1 (5.6)	29.5 (5.1)	29.9 (5.2)	29.4 (4.7)	<0.05
Current smoker, *n* (%)	181 (31.98)	109 (27.81)	77 (40.31)	45 (15.36)	60 (25.32)	472 (28.11)	<0.001
BADL disability, *n* (%)	37 (6.54)	45 (11.48)	49 (26.06)	42 (14.33)	94 (39.66)	267 (15.9)	<0.001
MMSE score, mean (SD)	25.47 (3.8)	24.02 (4.37)	22.56 (5.91)	23.5 (3.96)	20.34 (7.14)	23.8 (5.07)	<0.001
Charlson Index, mean (SD)	0.82 (1.33)	1.02 (1.45)	1.37 (1.71)	1.02 (1.56)	1.45 (1.9)	1.05 (1.55)	<0.001
Depression (GDS ≥ 5), *n* (%)	48 (9.76)	45 (12.71)	47 (27.01)	52 (19.11)	59 (29.21)	251 (16.8)	<0.001
PASE score, mean (SD)	115.61 (49.15)	80.65 (11.32)	21.53 (11.88)	53.27 (3.82)	28.74 (20.81)	73.6 (46.85)	<0.001
Δ in PASE, mean (SD)	−3.26 (18.24)	−5.64 (7.22)	5.92 (6.27)	0.87 (5.97)	−4.69 (3.74)	−2.25 (12.24)	<0.001

Data are presented as mean (SD) or *n* (%). Significant differences between men and women groups were analysed by Student's *t*‐test or *χ* ^2^ test. BADL, basic activity of daily living; BMI, body mass index; GDS, Geriatric Depression Scale; HPAC, high PA‐consistent; LPAD, low PA‐decreasing; LPAI, low PA‐increasing; MMSE, Mini‐Mental State Examination; MPAC, moderate PA‐consistent; MPAMD, moderate PA‐mildly decreasing; PA, physical activity; PASE, Physical Activity Scale for the Elderly; SD, standard deviation; Δ, change.

**Figure 1:**
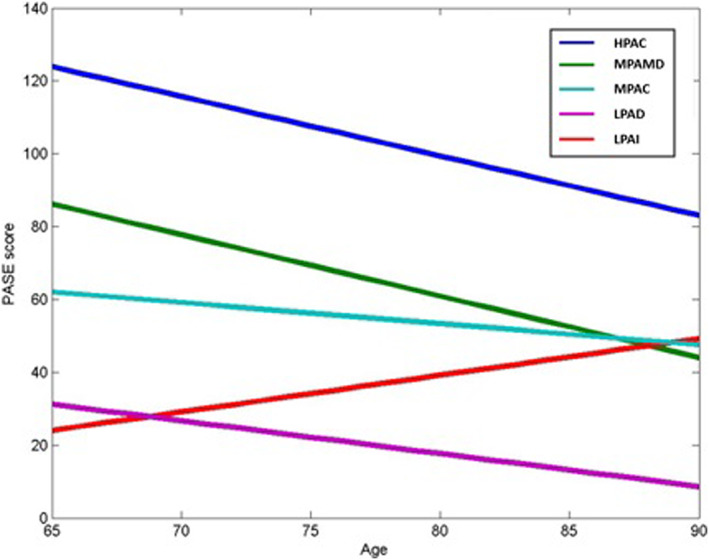
Physical activity (PA) trajectories by age. *N* = 1679; PA groups based on self‐reported PA via PASE scores. HPAC, high PA‐consistent; LPAD, low PA‐decreasing; LPAI, low PA‐increasing; MPAC, moderate PA‐consistent; MPAMD, moderate PA‐mildly decreasing; PASE, Physical Activity Scale for the Elderly.

Group‐based trajectory modelling yielded five PA trajectories as the best model: (i) high PA‐consistent (*n* = 566; 33.7%), (ii) moderate PA‐mildly decreasing (*n* = 392; 23.3%), (iii) low PA‐increasing (*n* = 237; 14.1%), (iv) moderate PA‐consistent (*n* = 191; 11.7%), and (v) low PA‐decreasing (*n* = 293, 17.5%) (*Figure* 1). The mean posterior probability of membership, an index of quality classification used in GBTM like ours, was 0.79 ± 0.65, indicating an acceptable classification of the subjects within each PA trajectory.

**Figure 2:**
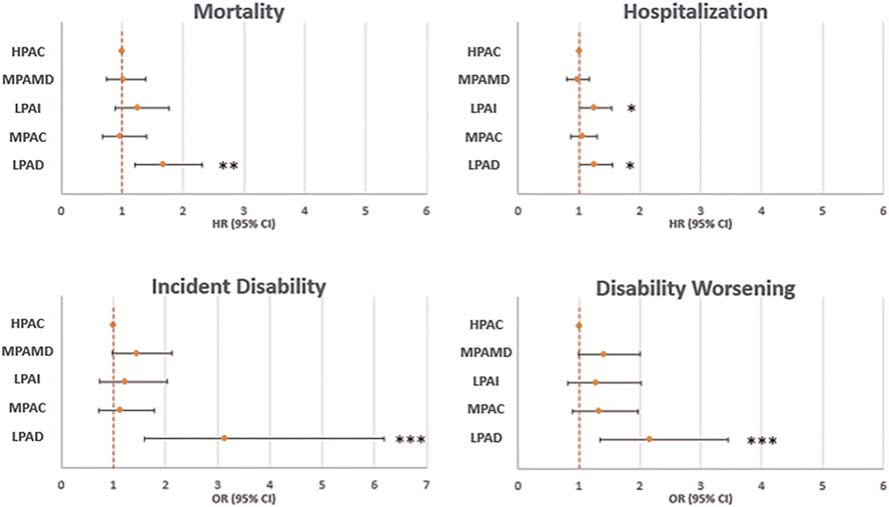
Forest plots of the risk of the different adverse outcomes in the different physical activity trajectory groups. CI, confidence interval; HR, hazard ratio; OR, odds ratio. ^*^ *P* < 0.05; ^**^ *P* < 0.01; ^***^ *P* < 0.001. HPAC, high PA‐consistent; LPAD, low PA‐decreasing; LPAI, low PA‐increasing; MPAC, moderate PA‐consistent; MPAMD, moderate PA‐mildly decreasing.

In the Cox regression model, subjects in the low PA‐decreasing trajectory group had a higher mortality risk than peers in the reference group (high PA‐consistent) across all models [hazard ratio (HR) 1.68; 95% confidence interval (CI) = 1.21–2.31 in the fully adjusted model (Model 3); *Table* 2, *Figure* 2].

**Table 2:** Multivariate associations between physical activity trajectories and adverse outcomes

	HPAC *n* = 566; 33.7%	MPAMD *n* = 392; 23.3%	LPAI *n* = 237; 14.1%	MPAC *n* = 191; 11.7%	LPAD *n* = 293; 17.5%
Mortality (HR; 95% CI)					
Raw model	*Reference*	1.41 (1.04–1.91)*	2.26 (1.62–3.14)***	1.27 (0.9–1.79)	5.15 (3.92–6.77)***
Model 1		1.19 (0.88–1.63)	1.56 (1.12–2.19)**	1.15 (0.8–1.64)	2.53 (1.86–3.44)***
Model 2		1.13 (0.83–1.54)	1.4 (0.99–1.97)	1.08 (0.76–1.55)	2.21 (1.62–3.03)***
Model 3		1.01 (0.74*–*1.39)	1.26 (0.89–1.78)	0.97 (0.68–1.4)	1.68 (1.21–2.31)**
Hospitalization (HR; 95% CI)					
Raw model	*Reference*	1.1 (0.92–1.32)	1.68 (1.37–2.05)***	1.19 (0.98–1.45)	2.22 (1.85–2.66)***
Model 1		1.04 (0.86–1.25)	1.43 (1.16–1.77)***	1.2 (0.98–1.47)	1.68 (1.37–2.07)***
Model 2		1.01 (0.84–1.21)	1.33 (1.08–1.64)**	1.15 (0.93–1.4)	1.48 (1.2–1.83)***
Model 3		0.97 (0.8*–*1.17)	1.24 (1.004–1.54)*	1.05 (0.86–1.3)	1.25 (1.01–1.55)*
Disability (OR; 95% CI)					
Raw model	*Reference*	1.84 (1.27–2.66)**	1.93 (1.21–3.09)**	1.66 (1.1–2.51)*	4.93 (2.62–9.28)***
Model 1		1.5 (1.02–2.2)*	1.5 (0.92–2.46)	1.23 (0.79–1.91)	3.39 (1.74–6.59)***
Model 2		1.51 (1.03–2.21)*	1.41 (0.86–2.32)	1.24 (0.8–1.94)	3.31 (1.7–6.48)***
Model 3		1.44 (0.97–2.13)	1.22 (0.73–2.04)	1.13 (0.72–1.78)	3.14 (1.59–6.19)***
Disability worsening (OR; 95% CI)					
Raw model	*Reference*	1.71 (1.22–2.38)**	1.99 (1.31–3.02)**	1.75 (1.21–2.52)**	3.33 (2.23–4.98)***
Model 1		1.46 (1.04–2.06)*	1.61 (1.05–2.49)*	1.48 (1.01–2.18)*	2.05 (1.3–3.18)**
Model 2		1.45 (1.03–2.04)*	1.52 (0.99–2.36)	1.47 (1.002–2.17)*	1.86 (1.19–2.91)**
Model 3		1.41 (0.99–2.01)	1.28 (0.81–2.02)	1.32 (0.89–1.97)	2.16 (1.35–3.45)**

Model 1 (age and gender); Model 2 (age, gender, and Charlson Index); Model 3 (age, gender, Charlson Index, Mini‐Mental State Examination, educational level, smoking status, Katz Index, and polypharmacy). CI, confidence interval; HPAC, high PA‐consistent; HR, hazard ratio; LPAD, low PA‐decreasing; LPAI, low PA‐increasing; MPAC, moderate PA‐consistent; MPAMD, moderate PA‐mildly decreasing; OR, odds ratio; PA, physical activity.

* *P* < 0.05.

** *P* < 0.01.

*** *P* < 0.001.

Subjects in the low PA‐increasing [HR 1.24; 95% CI = 1.004–1.54 (Model 3)] and low PA‐decreasing [HR 1.25; 95% CI = 1.01–1.55 (Model 3)] trajectory groups showed a significant increase in the likelihood of hospitalization when compared with subjects in the high PA‐consistent group, across all models (*Table* 2, *Figure* 2).

In the ‘raw’ model, logistic regression analysis showed a greater risk of progressing into incident disability across all the trajectories as compared with the reference high PA‐consistent group. These associations weakened as covariates were included in the models but remained significant for the low PA‐decreasing trajectory group [odds ratio (OR) 3.14; 95% CI = 1.59–6.19 (Model 3)].

The low PA‐decreasing trajectory group showed significant increased risk of worsening disability when compared with subjects classified into the high PA‐consistent group (OR 2.16; 95% CI = 1.35–3.45 (Model 3); *Table* 2, *Figure* 2).

We additionally sought to compare groups with similar baseline PA (similar starting risk) but divergent PA trends along time (low PA‐increasing vs. low PA‐decreasing trajectory groups). Subjects in the low PA‐decreasing trajectory group were more likely to be women (56% vs. 39%; *P* = 0.001), to be older (78.9 vs. 74.4 years; *P* < 0.0001), and to have difficulties in one or more BADLs at baseline (39.7% vs. 25.6%; *P* = 0.0032).

**Figure 3:**
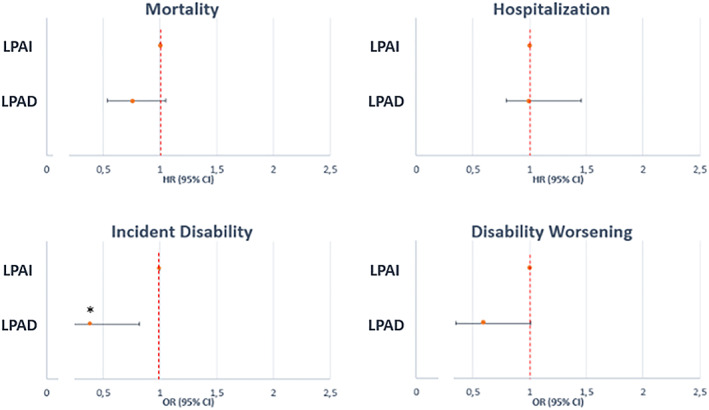
Forest plots of the differences in the risk of adverse outcomes between the low physical activity‐increasing and low physical activity‐decreasing trajectory groups. CI, confidence interval; HR, hazard ratio; OR, odds ratio. ^*^ *P* < 0.05; ^**^ *P* < 0.01; ^***^ *P* < 0.001. LPAD, low PA‐decreasing; LPAI, low PA‐increasing.

Taking the low PA‐decreasing as the reference, subjects in the low PA‐increasing trajectory showed a significantly lower risk for disability onset (OR 0.39; 95% CI = 0.19–0.82; *Figure* 3) in the fully adjusted model. We failed to find differences between these two trajectories in terms of mortality, hospitalization, or worsening disability risks.

Sensitivity analyses excluding subjects with posterior probabilities <0.5 (*n* = 65; 3.87% of the sample) for the group they were classified into did not meaningfully change the associations.^30^

## Discussion

The present study aimed at identifying PA trajectories in the TSHA and exploring their association with adverse outcomes. Overall, our study shows that in this large representative cohort of older adults, prospectively maintaining higher baseline PA levels at older ages is associated with a lower risk of relevant adverse outcomes (mortality, disability onset and worsening, and hospitalization), independently of important confounding factors. Subjects showing higher PA levels at baseline and sustaining them along time (high PA‐consistent trajectory) had lower odds of all adverse outcomes compared with those presenting with low baseline PA levels and an important decline along time (low PA‐decreasing trajectory) and lower risk for hospitalization than those with a low initial PASE score even when PA increased over time (low PA‐increasing trajectory). These findings support the need to promote effective intervention strategies aiming to maintain or increase PA among older adults, as a key factor for healthy aging.

We identified five longitudinal trajectories within our population. Of these, four showed a prospective reduction in PA at old age, which agrees with previous observations of a majority of older adults reducing PA along time.^48^ Of special note is the presence of one PA‐increasing trajectory (low PA‐increasing), suggesting the existence of qualitatively distinct trajectories in our population of interest, as previously described in other works,^49^, ^50^, ^51^, ^52^, ^53^, ^54^ and extending the findings of classical studies describing heterogeneous patterns of free‐living PA at older ages.^55^

Regarding the GBTM algorithm classification quality, the identified trajectory groups showed mean posterior probabilities of membership that were close to 0.8. As values of 0.7–0.8 are deemed indicative of an aggrupation that sufficiently discriminates between individuals with dissimilar patterns of change in a behaviour over time,^56^ we conclude that our classification is reliable.

In relation to the association of prospective PA changes and mortality, our results showing an increased risk for the low PA‐decreasing in relation to high PA‐consistent trajectory groups are consistent with the general notion that increased levels of PA lead to greater longevity in both men and women. These findings were also observed when using cross‐sectional PA estimates,^9^, ^20^, ^23^, ^25^, ^27^, ^28^, ^29^ prospective categorical changes in PA,^9^, ^33^, ^57^ and more recently, finite mixture modelling approaches for PA trajectories identification as the exposure variable,^39^ irrespective of the PA‐estimation tool (self‐reported vs. objective measures).^58^ While it is known that even a low level of participation in PA reduces mortality rates in comparison with inactive behaviour,^8^, ^27^, ^28^ the benefits increase in a dose–response manner at older ages in both men and women, with a saturation effect in the upper limit of both intensity and volume of PA.^23^ Interestingly, the magnitude of PA effect on mortality might be contingent upon the intensity of the displaced activity, which underscores the importance of this parameter as a determinant of PA effects on health.^59^

Importantly, the mortality‐sparing benefits of late‐life PA might only be slightly affected by pre‐cohort PA behaviour,^60^ meaning that at advanced age, even subjects that have never engaged in PA might gain similar benefits to those that used to be active. Additionally, PA seems to attenuate the excess risk of mortality associated with other classical predictors such as frailty,^61^ cognitive impairment,^62^ and poor mental, social, and physical health among elders.^63^

Because PA is a powerful countermeasureagainst the development and progression of chronic conditions, it is assumed toconstitute a means to reduce healthcare system utilization 64. In thiscontext, our results point to an important reduction in the likelihood ofhospitalization among those remaining active along time (High PA‐Consistenttrajectory) versus those showing low PA baseline levels (Low PA‐Increasing andLow PA‐Decreasing trajectories). The little research that is available hasdemonstrated that PA is related to fewer and shorter hospital admissions 65, 66.

With regard to the association between PA and disability onset, our results support previous work reporting an inverse association,^14^, ^67^ with greater PA levels (measured heterogeneously) associated with lower likelihood of functional disability captured as mobility,^67^, ^68^ clusters of BADL,^69^, ^70^ instrumental activities of daily living,^69^, ^70^ or both.^69^, ^70^, ^71^ The inverse PA–disability association seems to be graded, because slight increases in terms of volume^71^ and intensity^72^ are associated with significantly better functionality.

Yu *et al*. sought to explore associations between PA trajectories and functionality in a Taiwanese older adult cohort of 3186 subjects (mean age 63.89 ± 8.17 years; 50% women) using a growth mixture model along 11 years follow‐up.^73^ Notwithstanding the differences in methodology between their study and ours, the results were similar. Importantly, and analogous to our findings, their PA‐increasing trajectory group benefitted from protection against disability to a similar extent as those who remained highly active along follow‐up, despite their low baseline PA levels.^73^

Regarding the associations between PA levels and disability worsening, the limited evidence points to a negative association between higher activity levels (measured in diverse ways) and the odds of showing lower functionality at follow‐up. In this line, Tak *et al*. performed a meta‐analysis of four studies that reported associations between PA and BADL disability progression.^14^ These findings are also compatible with our data. Given the paucity of studies, further research is needed to study the complex relationships between PA and other determinants of disability.

The low PA‐increasing trajectory group showed an increased risk of hospitalization, but not disability, when compared with the high PA‐consistent group. We hypothesize that the strong association between increasing activity and functionality during late life might arise as the result of actual improvements in the physical domain of intrinsic capacity resulting from PA. Tellingly, associations between PA and functionality tend to be stronger in the case of motor activities of daily living,^54^, ^66^ whereas the associations with performance on more cognitive demanding tasks might be more modest.^69^, ^74^ On the other hand, the absence of significant reductions in hospitalization in the low PA‐increasing trajectory group observed here might suggest a limited potential of late‐life increases in PA to avoid outcomes that lead to hospitalization in elderly populations—mainly cardiovascular events, pulmonary disease exacerbations, and fractures resulting from lifelong development of prevalent conditions in the presence of low PA levels. Accordingly, members of more active trajectories are more likely to have been active during mild life, and thus, their odds of developing these conditions and subsequently being admitted to the hospital might be lower.

Our study has several strengths including the large sample size, excellent ascertainment of adverse outcomes, and the inclusion of relevant variables that could confound the associations. The TSHA is a representative population of community‐dwelling and institutionalized men and women with a wide range of ages and both. Furthermore, we used the novel GBTM, a powerful statistical tool to group subjects into qualitatively distinct developmental progressions that differ not only at baseline but also in the direction and magnitude of the change and that are not readily identifiable using *ad hoc*, *ex ante* classification rules. By doing so, we accounted for the factual dynamic nature of PA, overcoming bias of previous research. Although GBTM might remain unfamiliar for most of clinical focused researchers, it presents some compelling features that might be quite useful to study longitudinal data. The characterization of groups of subjects following different evolutions for a variable has commonly relied on subjective categorization based on clinical or empirical thresholds (cut‐points derived in other cohorts, tertiles, etc.). Although reasonable, these assignment rules present with some pitfalls. First, the existence of different groups is assumed a priori, and this point cannot be tested objectively. Second, they do not allow for assessing the precision of individual classifications to the various groups, and hence, uncertainty about individual group membership might emerge. In our case, an important strength of GBTM is that it allows for a data‐based in‐depth study of the features of potential healthy aging phenotypes (PA‐maintainers/increasers), which in turn could help clinicians to identify strategies for maintaining PA engagement among the elderly. To estimate PA levels, we used a well‐validated tool specific for older adults, whose advantages include its brevity, easy scoring process, the inclusion of activities other than exercise, and the inclusion of activities common to older ensuring a comprehensive assessment of overall PA. While objective PA measures are more reliable and overcome PA questionnaires' limitations, they might be impractical in large cohorts, as in ours. This work is one of the first to our knowledge trying to explore the presence of different PA trajectories within a cohort of older adults and their associations with important adverse outcomes in an older adult cohort.

Our study, nevertheless, has important limitations. First, self‐reported PA tools are poorly correlated with objectively measured PA, and their use is subject to the inclusion of recall errors and social desirability leading to bias, especially among older adults. Additionally, over‐reporting of PA levels, if present, would lead to an underestimation of the actual effect of PA on adverse outcomes.^7^, ^32^ Second, PASE cannot be translated into actual PA levels or the metabolic equivalent of task or time performing exercise. Thus, it fails at directly accounting for important modulators of the effect of PA on health, mainly intensity and volume. This makes our results challenging to interpret in relationship with other studies or PA recommendations. Third, hospitalization was only ascertained by checking Toledo Hospital Complex records. Although this centre is the reference hospital for the Toledo's province, where the whole sample dwelled at baseline, it is possible that some subjects have travelled or moved during follow‐up and some events could have gone unreported.

Although GBTM is increasingly been used for identifying trajectories for both exposures and outcomes in observational and experimental research, it is inherently limited in capturing individual variability and may lead to over‐grouping.^36^ Furthermore, we could only construct linear trajectories because we could only evaluate two time‐points prior to follow‐up.

Reverse causality cannot be excluded in our study, due to its observational nature. PA participation might be conditioned by health status and vice versa. Nevertheless, the associations remained significant after adjustment for important risk factors, co‐morbidities, and functional status.

We acknowledge the potential absence of unrecognized or uncontrolled covariates that could affect our observations. Of note is the absence of a measure of sedentary behaviour as a competing exposure. Sedentary behaviour has recently been postulated as a key health factor, independent of PA patterns, and is associated with mortality,^75^, ^76^ disability,^69^, ^77^ and increased health care expenditure.^78^ Furthermore, as we did not have information about PA before the study entry time, we are not able to assess whether the benefit from PA observed in this study could respond to a lifelong acquired benefit rather than to a recently gained PA beneficial effect. Direct comparison of our study with others is not possible due to the unique distribution of trajectories that GBTM yields within individual populations. Finally, our sample is restricted to a specific area of Spain with unique features. Although this fact may limit the generalizability of our results, it offers the advantage of reduced potential confounding by race, education, social economic class, and access to health care.

Our study contributes to expand the notion of PA as a powerful modifiable factor that promotes healthy aging by means of preventing important adverse outcomes including mortality, disability, and hospitalization. It does so from a new perspective, by using a novel approach that allows to empirically identify different PA‐related aging phenotypes, that as our results suggest, are associated with relevant outcomes for older people. The prevention of BADL disability is especially relevant, because it is associated with receiving home care services and an increased risk of long‐term nursing home admission and health care costs.^14^ In the context of a global aging population, our findings have important clinical and economic implications. Given that the association between PA and healthy aging seems to be valid and physiologically plausible, especially if the PA is in the form of structured exercise, the goal should be to enhance exercise participation among older people living in the community. Unfortunately, PA levels fall dramatically in the last years of life.^20^ If, as the evidence indicates, any activity above sedentarism in terms of volume^8^, ^27^, ^28^, ^29^, ^35^ and intensity,^23^, ^32^, ^59^ and longitudinally maintaining PA levels, are associated with better health outcomes in older adults,^27^, ^29^, ^79^ clinicians, relatives, and caregivers should encourage them to be as active as possible, both during planned exercise sessions and in everyday life activities. Policymakers, for their part, should increase social awareness and promote accessible, popular, and everyday activities such as walking and active commuting among older populations. Hopefully, efforts are being made to increase late‐life PA participation.^80^

In summary, we confirmed our hypotheses in our older adult cohort that consistent high PA levels provide protection against important adverse outcomes when compared with low PA baseline levels and decreasing PA. Importantly, increasing PA levels during late life might entail a lower risk of disability in comparison with prospectively reducing activity.

## Conflict of interest

The authors declare that they have no conflict of interest.

## Ethical issues

The study protocol was approved by the Clinical Research Ethics Committee of the Toledo Hospital, Spain. This work was performed according to the ethical standards laid down in the 1964 Declaration of Helsinki and later amendments. Participants signed informed consent forms prior to their inclusion in the cohort. The authors certify that they comply with the ethical guidelines for authorship and publishing of the *Journal of Cachexia, Sarcopenia and Muscle*.^81^

## Supporting information


**Table S1.** Baseline characteristics of included vs. not‐included subjects
