# Supplementary material for: Physical activity trajectories, mortality, hospitalization, and disability in the Toledo Study of Healthy Aging
Source: J Cachexia Sarcopenia Muscle. 2020 Mar 12;11(4):1007–17. doi: 10.1002/jcsm.12566 (PMC7432572; doi:10.1002/jcsm.12566)
Supplement: Supplementary file 1 — Table S1. Baseline characteristics of included vs. not‐included subjects [file JCSM-11-1007-s001.docx]

|  | **Included**  **n= 1679** | **Not included**  **n= 809** | **Between groups differences (p-value)** |
| --- | --- | --- | --- |
| Age, mean (SD) | 74.94 (5.06) | 77.34 (7.29) | <0.001 |
| Men, No. (%) | 701 (41.74) | 391 (48.33) | <0.001 |
| BMI, mean (SD), kg.m^-2^ | 29.4 (4.7) | 28.92 (5.39) | <0.05 |
| Current smoker, No. (%) | \| 472 (28.11) \| \| --- \| | 278 (34.36) | <0.01 |
| BADL disability, No. (%) | 267 (15.9) | 237 (30.58) | <0.001 |
| MMSE score, mean (SD) | 23.8 (5.07) | 21.26 (7.92) | <0.001 |
| Charlson Index, mean (SD) | 1.05 (1.55) | 1.49 (1.92) | <0.001 |
| Depression (GDS ≥ 5), No. (%) | 251 (16.8) | 148 (22.35) | <0.001 |
| PASE score, mean (SD) | 73.6 (46.85) | 52.33 (47.78) | <0.001 |
| ∆ in PASE, mean (SD) | -2.25 (12.24) | -1.19 (8.94) | <0.001 |

**SUPPLEMENTARY TABLE 1. BASELINE CHARACTERISTICS OF INCLUDED vs. NOT-INCLUDED SUBJECTS**

Data are presented as mean (SD) or No. (%). Significant differences between men and women group were analyzed by Student’s t-test or Chi^2^ test.

HPAC: High PA-Consistent; MPAMD: Moderate PA-Mildly Decreasing; MPAC: Moderate PA-Consistent; LPAD: Low PA-Decreasing; LPAI: Low-PA Increasing; BMI: Body Mass Index; BADL: Basic Activitie+ental State Examination; GDS: Geriatric Depression Scale; PASE: Physical Activity Scale for the Elderly. ∆: Change.
